# Mechanisms of Peripheral and Central Pain Sensitization: Focus on Ocular Pain

**DOI:** 10.3389/fphar.2021.764396

**Published:** 2021-11-30

**Authors:** Giulia Puja, Balazs Sonkodi, Rita Bardoni

**Affiliations:** ^1^ Department of Life Sciences, University of Modena and Reggio Emilia, Emilia-Romagna, Italy; ^2^ Department of Health Sciences and Sport Medicine, University of Physical Education, Budapest, Hungary; ^3^ Department of Biomedical, Metabolic and Neural Sciences, University of Modena and Reggio Emilia, Emilia-Romagna, Italy

**Keywords:** cornea, trigeminal ganglion, peripheral and central sensitization, synaptic transmission, descending modulation, ocular pain

## Abstract

Persistent ocular pain caused by corneal inflammation and/or nerve injury is accompanied by significant alterations along the pain axis. Both primary sensory neurons in the trigeminal nerves and secondary neurons in the spinal trigeminal nucleus are subjected to profound morphological and functional changes, leading to peripheral and central pain sensitization. Several studies using animal models of inflammatory and neuropathic ocular pain have provided insight about the mechanisms involved in these maladaptive changes. Recently, the advent of new techniques such as optogenetics or genetic neuronal labelling has allowed the investigation of identified circuits involved in nociception, both at the spinal and trigeminal level. In this review, we will describe some of the mechanisms that contribute to the perception of ocular pain at the periphery and at the spinal trigeminal nucleus. Recent advances in the discovery of molecular and cellular mechanisms contributing to peripheral and central pain sensitization of the trigeminal pathways will be also presented.

## Introduction

Ocular pain is produced by stimulation of primary sensory neurons at the eye surface or by alterations along the ocular pain pathway. Peripheral and central sensitization at these levels is fundamental for the development of long lasting pain perception.

At the ocular surface, the cornea represents the most innervated and sensitive tissue. Its innervation is supplied exclusively by small myelinated and unmyelinated sensory fibers, which are located between the different layers of the corneal epithelium, protecting cornea integrity from potential injuries. Corneal sensory fibers are mainly associated with pain*:* psychophysical studies in humans have demonstrated that corneal mechanical, chemical or thermal stimulation produces aversive or nociceptive sensations (Kenshalo et al., 1960; Beuerman and Tanelian, 1979; Belmonte et al., 1999), except for the purely cold sensations provoked by low-temperature stimuli of moderate intensity (Acosta et al., 2001). Direct activation of corneal nerve terminals evokes also protective reflexes, such as eye blinks, tear formation, endocrine and cardiovascular responses (Bereiter et al., 1996; Boscan and Paton, 2002).

**FIGURE 1:**
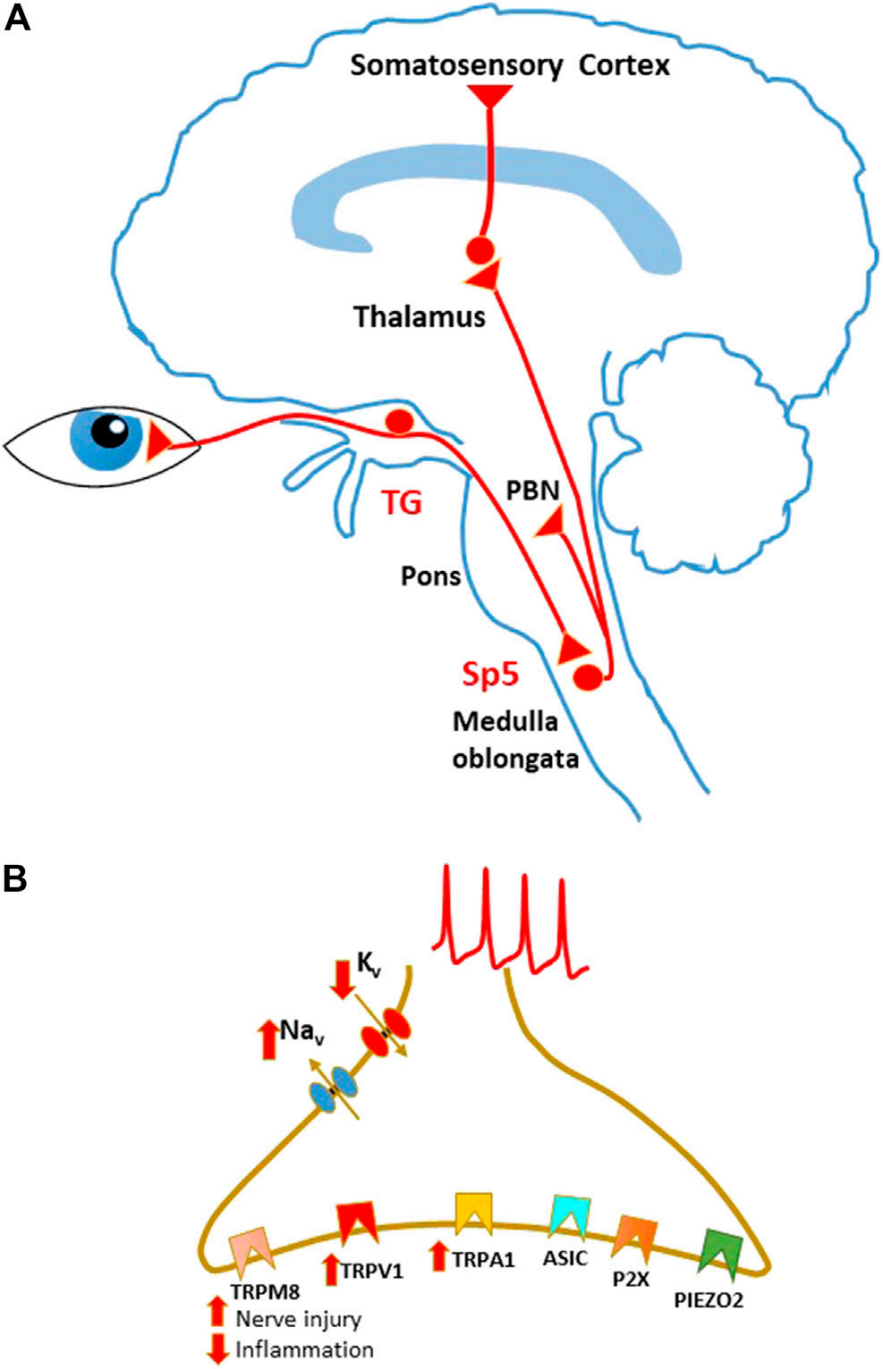
Schematic representation of sensory pathways involved in corneal pain transmission. **(A)** Sensory pathways conveying corneal nociceptive input to the central nervous system. Corneal sensory input is transmitted by corneal nociceptors, whose cell bodies are located in the trigeminal ganglion (TG). Central terminals of nociceptors project to the spinal trigeminal nucleus (Sp5) in the brain stem. Projection neurons in these regions send ascending pathways to several areas, including the parabrachial nucleus (PBN) and the thalamus, that in turn project to higher centers. **(B)** Principal ion channels involved in corneal sensory transduction on the nociceptor peripheral terminals. During peripheral sensitization, TRPV1 and TRPA1 are usually upregulated, while TRPM8 function is enhanced in neuropathic pain and decreased during inflammation.

Primary afferent fibers innervating the cornea belong to the myelinated Aδ and unmyelinated C type and run in the trigeminal nerve (ophthalmic branch, V1), whose ganglion (trigeminal ganglion, TG) contains the somas of the primary sensory neurons. The central branches of corneal afferents reach the trigeminal spinal nucleus (Sp5) in the brainstem, where they contact the second order sensory neurons, represented by both projection and local circuit neurons (Figure 1A).

Corneal nerve structure and function are adversely affected by many ophthalmic and systemic disorders. Persistent ocular pain can be provoked by a long-lasting noxious stimulus or damage to the ocular surface (nociceptive pain) or can result from abnormalities in the ocular neurosensory apparatus itself (neuropathic pain). Persistent and abnormal activation of corneal nociceptors can lead to pain sensitization, occurring at peripheral and/or central sites and manifesting as spontaneous pain, hyperalgesia (increased response to a noxious stimulus), and allodynia (pain evoked by a normally innocuous stimulus) (Galor et al., 2018; Guerrero-Moreno et al., 2020).

While several mechanisms of pain sensitization occurring at the peripheral corneal afferent endings have been identified, synaptic alterations affecting second order neurons in the spinal trigeminal nucleus have not been fully investigated. On the other hand, new technical approaches developed during the last decade (such as neuronal genetic labelling and optogenetic stimulation) have provided a better comprehension of the trigeminal circuits involved in sensory transmission and pain sensitization. In this review, we will outline recent advances in understanding corneal pain processing, with particular focus on the mechanisms leading to pain sensitization.

## Peripheral Mechanisms Mediating Ocular Pain

### Types of Corneal Sensory Fibers

Nociceptors innervating the corneal surface can be classified as mechanonociceptors, polymodal nociceptors and cold-sensitive receptors (Gallar et al., 1993; Belmonte et al., 2004; Gonzalez-Gonzalez et al., 2017).

Mechanonociceptors (MNs) represent about 10% of corneal fibers (mostly Aδ type) and are activated exclusively by noxious mechanical forces generated by external objects, presence of foreign bodies, air pressure or distortion of epithelium layer caused by drying ocular surface. Their thresholds are low in comparison with the MNs in the skin: however, since their endings are very close to corneal surface, they are probably excited by similar actual forces (Boada, 2013). MNs respond to mechanical stimulation mainly with a short lasting, phasic discharge of action potentials (APs), thus encoding the dynamic changes of the stimulus (Belmonte and Giraldez, 1981; Belmonte et al., 1991).

A large population of corneal sensory fibers (about 40%) are polymodal nociceptors (PNs). They respond with a strong discharge in response to a broad spectrum of stimuli: mechanical energy near or above noxious level, heat (>39°C) or noxious cold, exogenous chemical liquid or gaseous irritants, bacterial toxins. PNs can be also activated by endogenous chemical mediators released by damaged corneal tissue or deriving from plasma leaking from limbal vessels. Most PNs belong to C-type fibers and generate an irregular, continuous discharge, providing information about the intensity of the stimulation (Belmonte and Giraldez, 1981; Belmonte et al., 1991; Chen et al., 1997).

About 50% of corneal afferent fibers are represented by cold-sensitive receptors (CRs), including both Aδ and C fibers. They are activated by cooling of the corneal surface (induced by corneal application of cold solution or cold air or by tear film evaporation) or by the increase of tear osmolarity (Belmonte and Giraldez, 1981; Acosta et al., 2001; Parra et al., 2014). CRs fire tonically and contribute to maintain ocular surface wetness by regulating basal tear flow and blinking rate (Hirata and Meng, 2010; Hirata et al., 2012). Based on their activation threshold, CRs can be divided in low and high threshold (LT and HT). LT receptors discharge spontaneously at rest and increase their firing rate under small decreases of the corneal temperature below the normal value (about 34–35°C), providing a sensation of cold and dryness. HT receptors, whose activation causes a sensation of dryness and pain, do not show spontaneous activity at normal corneal temperature (they remain silent at temperatures >29°C) and are activated only by strong cooling (Hirata et al., 2012; Hirata and Rosenblatt, 2014).

### Membrane Channels Involved in Corneal Sensory Transduction

Corneal nociceptors are involved in sensory transduction, that is mediated by several classes of ion channels, detecting different types of nociceptive stimuli. Opening of these channels on the nociceptor peripheral terminals generates an inflow of cations, which mediates membrane depolarization. Supra-threshold depolarizations, in turn, activate sodium and potassium voltage-dependent channels, generating APs that propagate to the neuronal soma in the TG and to the central terminals in Sp5 (Figure 1B).

Corneal transduction of mechanical stimuli is mainly performed by PIEZO2 channels, large membrane proteins with a homotrimeric propeller-shaped structure, comprising a central ion-conducting pore module and three peripheral mechanosensing blades with 38 transmembrane domains. Mechanical activation of this channel generates a cationic current, that depolarizes excitable cells (Coste et al., 2010; Coste et al., 2012). PIEZO2 is mainly activated by innocuous mechanical forces and is expressed in dorsal root ganglia (DRG) and TG sensory neurons, in tactile epithelial Merkel cells, and in the sensory endings of proprioceptors (Woo et al., 2014; Woo et al., 2015). Accordingly, mice carrying a conditional deletion of PIEZO2 in sensory neurons or in Merkel cells show severe deficits in tactile discrimination and movement coordination, while responses to mechanical nociceptive stimulation are unaffected or only partially diminished (Woo et al., 2014; Woo et al., 2015; Murthy et al., 2018). Recent experimental evidence suggests that PIEZO2 is also involved in different forms of mechanoceptive sensitization, such as mechanical allodynia, generated by inflammation or nerve injury (Murthy et al., 2018; Szczot et al., 2018).

In the cornea, PIEZO2 is expressed by pure MN sensory neurons and by a subpopulation of PNs (Bron et al., 2014; Fernandez-Trillo et al., 2020). In sensory-specific PIEZO2 knock out mice, electrophysiological responses to mechanical stimulation of corneal MNs and PNs were significantly reduced and the eye blink reflex was impaired (Fernandez-Trillo et al., 2020). The expression of highly sensitive mechanotransducing channels like PIEZO2 on corneal nociceptors is critical for the early detection of low-intensity mechanical stimuli, potentially harmful to the corneal epithelium.

PIEZO2 has been also identified as the principal mechanotransduction channel for proprioception (Woo et al., 2015), however strong evidence is lacking that corneal trigeminal afferents and extraocular muscle spindles contribute to proprioception (Weir et al., 2000; Rao and Prevosto, 2013). Nevertheless, it was suggested that the primary afferents of extraocular muscle spindles initiate the corneal reflex (Bratzlavsky, 1972). Under neuropathic cornea disease, somatosensory PIEZO2 channels could be microinjured mechano-energetically and could alter genetically preprogrammed reflexes with longitudinal central nervous system consequences. The repetitive reinjury of PIEZO2 channels could cause chronic pain even in the absence of secondary harsher tissue injury (Sonkodi et al., 2021a; Sonkodi et al., 2021b).

Both TRPV1 and TRPA1 channels, belonging to the transient receptor potential (TRP) family of ion channels, are involved in corneal pain transduction. All TRP channels possess a tetrameric structure, where each monomer consists of six transmembrane domains (S1-S6). A pore loop, located between S5 and S6, forms the permeation pathway to cations. In the cornea, peptidergic PNs highly express TRPV1, that is directly activated by heat, protons, and high osmolarity (Murata and Masuko, 2006; Zhang et al., 2007; Hegarty et al., 2014; reviewed in: Mergler et al., 2014; Luo et al., 2021). A subpopulation of PNs present the TRPA1 channels, responding to exogenous irritants, toxins, chemicals, strong cold, and endogenous agents (such as ROS and lipid peroxidation products) (Acosta et al., 2014; Schecterson et al., 2020). In DRG neurons heteromeric interactions between TRPV1 and TRPA1 have been reported (Akopian, 2011). Interestingly, physical association between TRPA1 and TRPV1 is regulated by the membrane adaptor protein Tmem100: when this protein is present, TRPV1 mediated inhibition on TRPA1 is reduced. This leads to the potentiation of TRPA1 activity, contributing to persistent pain (Weng et al., 2015). Although the co-expression of TRPV1 and TRPA1 in corneal sensory neurons is still debated (Gonzalez-Gonzalez et al., 2017; Schecterson et al., 2020), the presence of TRPV1-A1 complexes in corneal afferents could play an important role in pain transduction and sensitization. Beside TRPV1 and TRPA1, other channels are involved in sensory transduction in corneal PNs. These include ASICs (acid-sensing ion channels, opened by protons) and P2x (purinergic ionotropic receptors binding ATP) (Belmonte et al., 2015; Belmonte, 2019).

TRPM8 channel, another member of the TRP receptor family, is highly expressed by corneal CRs, where it is sensitive to dynamic downward shifts of temperature and to moderate osmolarity increases (Parra et al., 2010; Parra et al., 2014; Quallo et al., 2015). Additional channels contributing to cold transduction include background potassium channels, closed by cooling of the corneal surface, thereby inducing membrane depolarization and AP firing (Viana et al., 2002) and potassium K_v_1 channels, whose opening sets the threshold of CR activation and counteracts the cold-induced response in PNs (Madrid et al., 2009).

### Molecular Mechanisms of Peripheral Pain Sensitization

Beside sensory transduction in acute ocular pain, corneal nociceptors are also involved in several forms of peripheral sensitization, which develop during prolonged exposure to painful stimuli. Peripheral sensitization is defined as the increased responsiveness and reduced threshold of nociceptive neurons in the periphery of the sensory system, induced by local inflammation or by peripheral nerve injury.

In the cornea, several conditions can lead to tissue inflammation: infections caused by bacteria, viruses or fungi; eye injuries; exposure to irritant chemicals or ultraviolet radiation (UV); tear evaporation and hyperosmolarity in dry eye disease (DED). Damaged corneal tissue and immune cells release several molecules and inflammatory mediators, such as ATP, H^+^, Substance P (SP), Neurokinin A, Tumor necrosis factor alpha (TNF-α), prostaglandin E2 (PGE2), and interleukins (ILs), which interact with membrane receptors/channels of nociceptor ending membrane. This may lead to the opening and/or modifications of ion channels involved in sensory transduction (directly or by activating intracellular pathways), depolarization of nerve endings, increase of nociceptor excitability, and spontaneous firing. Consistently, several electrophysiological studies have demonstrated that peripheral corneal nerves sensitize whenever exposed to inflammatory milieu or to DED conditions (Gallar et al., 2007; Kurose and Meng, 2013; Parra et al., 2014).

Peripheral sensitization is observed also in case of damage to corneal nerve fibers, leading to neuropathic pain. Corneal nerve injuries can be generated by several factors or disorders, including photorefractive surgeries, DED, cornea abrasion, chemicals, radiations, diabetes, autoimmune diseases (such as the Sjögren’s syndrome), fibromyalgia, herpes zoster, and systemic medications. Injury of the corneal nerve induces initially a reduced or total loss of sensitivity of the damaged area, determining insensitivity or higher threshold to natural stimuli in the injured axons (Beuerman and Schimmelpfennig, 1980; Lee et al., 2002; Gallar et al., 2004; Cho et al., 2019). The subsequent regeneration of some damaged axons determines the formation of neuromas (i.e. axons surrounded by connective tissue and immune cells) and accumulation of ion channels in the neural stumps (Lisney and Devor, 1987; Devor et al., 1993). This can lead to an aberrant function of the peripheral nerve endings, generating spontaneous impulse bursts in absence of stimulation (ectopic activity) and/or paroxysmal firing in response to mild mechanical and chemical stimuli (Rivera et al., 2000; Luna et al., 2021).

Molecular mechanisms of sensitization involving ion channels and receptors expressed by peripheral trigeminal fibers have been thoroughly investigated by using several animal models of ocular pain (Table 1) (reviewed in Belmonte et al., 2015, Belmonte, 2019; Goto et al., 2016; Andersen et al., 2017; Guerrero-Moreno et al., 2020). We will present here some of the most recent studies, which have added interesting insight to this topic.

**TABLE 1:** Summary of experimental approaches used to induce and study ocular pain in animals.

Experimental procedure	Ocular pain model	References
Chemical (saline, mustard oil, capsaicin, CO_2_ application), thermal, mechanical or electrical corneal stimulation	Acute corneal pain	Lasagni-Vitar et al. (2021), Bereiter and Bereiter (1996), Meng and Bereiter (1996), Martinez and Belmonte (1996), Meng et al. (1997), Meng et al. (1998), Hirata et al. (2000), Hirata et al. (2004), Khalilzadeh and Saiah (2017)
Acetic acid application to ocular surface	Corneal irritation and acute corneal pain.	Martinez and Belmonte (1996)
Topical application of benzalkonium chloride	Ocular surface inflammation	Byun et al. (2020)
Alkali burn (NaOH application on cornea)	Inflammatory and neuropathic pain	Xiang et al. (2017)
Corneal ultraviolet irradiation	Photokeratitis Corneal inflammation	Tashiro et al. (2010); Acosta et al. (2014).
Endotoxin/Lipopolysaccharide (LPS) on cornea surface	Uveitis Intraocular inflammation	Bereiter et al. (2005)
Excision of lacrimal glands	Dry eye disease (DED) Inflammatory and neuropathic pain	Rahman et al. (2015), Hatta et al. (2019), Li et al. (2019), Fakih et al. (2019), Fakih et al. (2021)
Corneal surgical lesion	Corneal refractive surgery. Inflammatory and neuropathic pain.	Luna et al. (2021)
Controlled cutting of stromal nerve fibers	Corneal nerve damage. Neuropathic pain.	Zhang et al. (2012)

TRPV1 and TRPA1 channels undergo important changes during persistent corneal pain. *De novo* channel expression, increase of membrane trafficking and channel phosphorylation have been reported in corneal pain of both inflammatory and neuropathic origin, causing the potentiation of channel function and the increase of membrane depolarization. In an experimental model of keratitis induced by ultraviolet (UV) radiation, nocifensive responses produced by application of capsaicin and AITC (TRPV1 and TRPA1 agonists, respectively) were potentiated in irradiated eyes compared to controls (Acosta et al., 2014). In a rat model of DED (the excision of the lacrimary glands), TRPV1-mediated effects on ongoing activity and sensitivity to heat of corneal nociceptors were increased (Hatta et al., 2019). Finally, the upregulation of TRPV1, TRPA1, ASIC1, and ASIC3 mRNA was detected in the ophthalmic branch of the trigeminal nerve in a mouse model of severe DED caused by the excision of Harderian and extraorbital lacrimal glands (Fakih et al., 2021).

Voltage-dependent sodium channels (Na_v_) are actively involved in corneal nociceptor sensitization: perfusion with amitriptyline (a Na_v_ channel blocker) was less effective in tear-deficient mice, suggesting the occurrence of changes in the expression of these channels induced by ocular dryness (Masuoka et al., 2018). In guinea pig excised eyes, previously subjected to a corneal surgical lesion, AP discharges of PNs were increased in response to chemical corneal stimulation (CO_2_ application) (Luna et al., 2021). Similarly, removal of the main lachrymal gland in guinea pigs enhanced the ongoing AP firing and the responses to cooling of corneal CRs. These effects were mediated by the increase of the sodium currents and the decrease of potassium currents in TG neurons (Kovacs et al., 2016). In a different study, acute treatment of corneal nociceptor endings with the pro-inflammatory substances TNF-α and IL-1β increased the functional availability of Na_v_ channels at the terminal. This caused a shift of the spike initiation zone toward the axonal end, increasing the nociceptor excitability. Interestingly, the same effect on the spike initiation zone was observed in an animal model of photokeratitis induced by UV exposure (Goldstein et al., 2019).

TRPM8 channels undergo different changes depending on the type of corneal injury (Belmonte, 2019). TRPM8 function in mouse cold sensitive corneal fibers was inhibited by perfusion of inflammatory mediators (such as bradykinin, PG, histamine), which caused a reduction of ongoing cold-evoked impulse activity, recorded *in vitro* (Zhang et al., 2012). In contrast, corneal nerve injury increased the functional expression of TRPM8 in CRs, enhancing their cold sensitivity and causing a rise in the ongoing firing activity and basal tearing (Piña et al., 2019). Removal of lacrimatory gland in mice enhanced the TRPV1 expression in corneal TRPM8+ fibers, leading to increased AP firing in response to cold and to cold allodynia (Li et al., 2019).

Corneal nociceptor terminals express neuropeptides, in particular SP and CGRP (calcitonin-gene-related peptide) (Murata and Masuko, 2006). Following corneal injury, performed through superficial epithelial abrasion, CGRP expression in peripheral nociceptor terminal was upregulated (Hegarty et al., 2018). Release of SP and CGRP exerts a proinflammatory action (defined as “neurogenic inflammation”), by promoting the release of other inflammatory mediators, cell chemotaxis, and plasma extravasation. Consistently, ocular surface inflammation in rats, induced by topical application of 0.1% benzalkonium chloride, enhanced the expression of SP in trigeminal neurons (Byun et al., 2020), while ablation of the SP gene Tac1 or blockade of SP receptor NK1 reduced ocular nociceptive responses in mice, induced by saline application (5 M NaCl) on corneal surface (Lasagni Vitar et al., 2021).

A recent study suggests that cornea epithelial cells actively participate to peripheral pain sensitization. Indeed, TRPV-4 channels expressed on these cells can act as osmotic and thermal sensors: heat or cell swelling, induced by cell hypotonicity, trigger the opening of these channels, determining calcium influx, ATP release and modulation of corneal sensory fibers (Lapajne et al., 2020).

## Central Mechanisms of Ocular Pain

### Eye Pain Processing in the Trigeminal Spinal Nucleus (Sp5)

**FIGURE 2:**
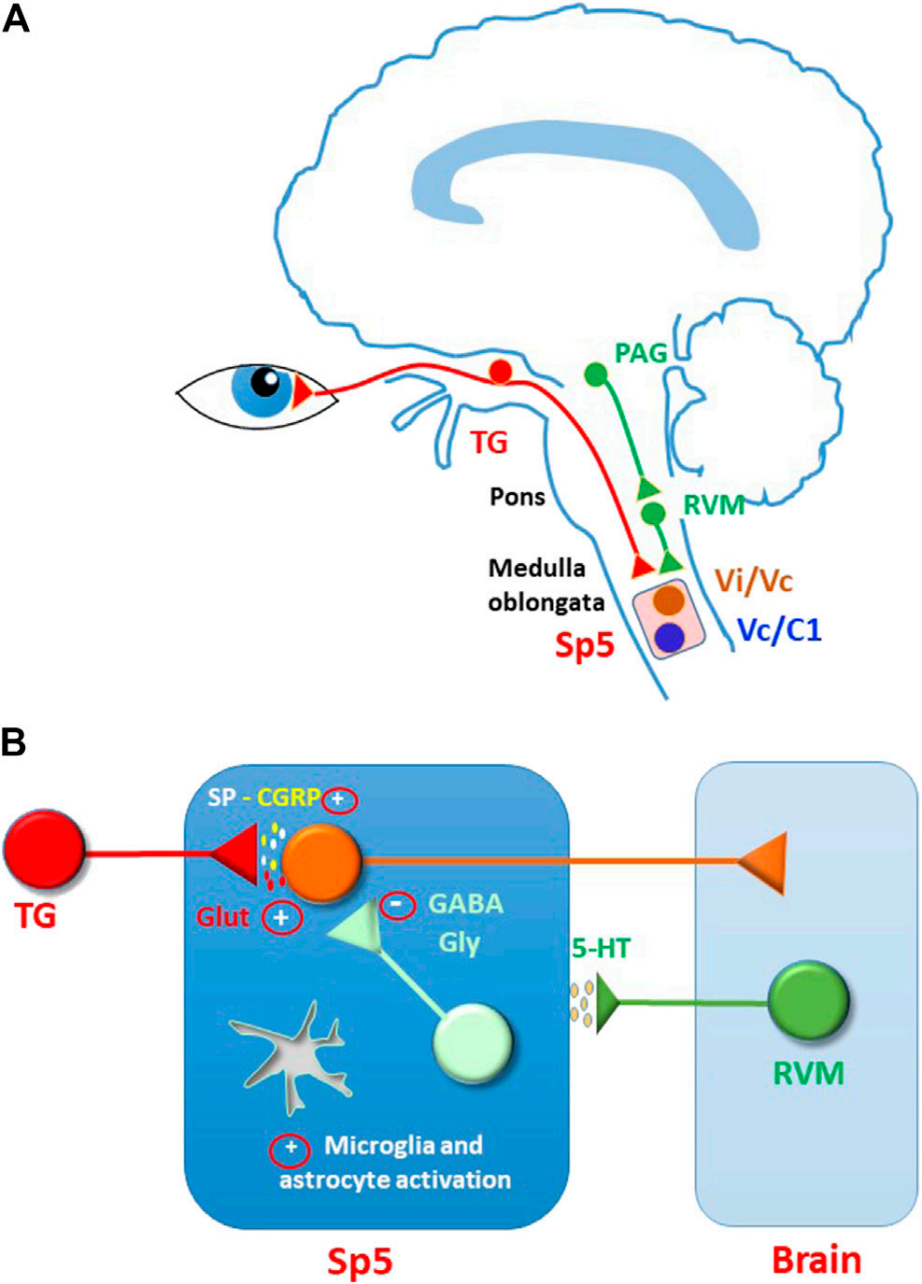
Modulation and sensitization of sensory input in the spinal trigeminal nucleus (Sp5). **(A)** Corneal sensory input is processed in Sp5, mainly at the transition between the subnuclei interpolaris and caudalis (Vi/Vc) and at the junction between the subnucleus caudalis and the upper cervical spinal cord (Vc/C1). Sp5 activity is controlled by descending modulation, comprising serotoninergic pathways. Serotoninergic neurons are located in rostral ventral medulla (RVM) and are activated by projection neurons in periaqueductal grey area (PAG). **(B)** Hypothetical mechanisms sustaining central ocular pain sensitization in Sp5. Persistent corneal nociceptive input may induce a general increase of synaptic excitation (mostly mediated by glutamate and peptides) and a decrease of synaptic inhibition (mediated by GABA and glycine). As reported for several forms of spinal and trigeminal pain, glutamate receptors could be potentiated by increased phosphorylation and participate to plasticity phenomena, such as wind-up and LTP. Synaptic inhibition could be depressed through changes of chloride equilibrium potential, LTD, neuronal loss, decrease of transmitter release, and presynaptic facilitation. Furthermore, a switch in the function of serotoninergic modulation from anti-to pro-nociceptive could contribute to the hyperexcitability state. Further studies are needed to confirm these mechanisms in the ocular pain system.

Corneal sensory input is transmitted from peripheral terminals through the TG and along the central terminals to the brain stem. Initial processing of the sensory information occurs in the trigeminal spinal nucleus (Sp5), located in the medulla oblongata. This nucleus consists of three subnuclei (oralis, interpolaris, caudalis), the most caudal of which, the subnucleus caudalis, extends into the cervical spinal cord. Two regions are particularly involved in the processing of corneal pain: the transition between the subnuclei interpolaris and caudalis (Vi/Vc) and the junction between the subnucleus caudalis and the upper cervical spinal cord (Vc/C1) (Marfurt and Del Toro, 1987) (Figure 2A). Beside receiving sensory inputs from several cranio-facial structures, these areas are also connected to each other by intersubnuclear projections (Nasution and Shigenaga, 1987; Jacquin et al., 1990).

Ocular stimulation (mechanical, chemical or electrical) activates trigeminal nerve sensory fibers, carrying information to Vi/Vc and Vc/C1 neurons: corneal stimulation or intravitreal capsaicin induced a bimodal distribution of the cFos gene expression (a marker for intense neural activation), showing a rostral peak in Vi/Vc and a caudal peak in Vc/C1 (Lu et al., 1993; Strassman and Vos, 1993; Bereiter and Bereiter, 1996; Martinez and Belmonte, 1996; Meng and Bereiter 1996).

*In vivo* electrophysiological recordings from Vi/Vc and Vc/C1 in rats have identified different subpopulations of neurons, exhibiting specific properties in response to cornea stimulation. In general, neurons receiving a corneal input are for the vast majority nociceptor-specific, activated either by corneal nociceptors only or by convergent corneal and cutaneous nociceptors (Meng et al., 1997; Meng et al., 1998; Hirata et al., 1999; Hirata et al., 2004).

In the Vi/Vc, two neuronal classes have been described:

- Type I: include both corneal specific units and neurons receiving also convergent cutaneous inputs. A late excitation is evoked in these neurons in response to corneal stimulation by CO_2_.
- Type II: represented only by neurons with convergent corneal and cutaneous inputs. These units are subjected to strong feedforward inhibition, since they respond to corneal stimulation with an inhibitory phase followed by late excitation. This class includes also neurons responding to acute changes of moisture status of ocular surface, importantly involved in the reflex of lacrimation.

Differently from Vi/Vc, all Vc/C1 neurons belong only to the Type I class and show convergent receptor fields.

Responses evoked by corneal electrical stimulation in Vi/Vc and Vc/C1 neurons are differently modulated by opioids: while all Vc/C1 units are inhibited by morphine, the responses of many Vi/Vc neurons are enhanced by *μ* receptor (MOR) agonists (Meng et al., 1998; Hirata et al., 1999). Interestingly, local administration of morphine to Vc/C1 region increased the responses to CO_2_ in Vi/Vc, confirming the presence of intersubnuclear connections that could contribute to opioid analgesia in corneal pain (Meng et al., 1998; Hirata et al., 2000).

All these results demonstrate that the ophthalmic division of the trigeminal nerve provides a dual sensory representation of the cornea in the Sp5. As pointed out by Bereiter et al. (2000), this redundancy may be explained by different roles played by Vi/Vc and Vc/C1 regions in the sensory elaboration of corneal pain. The properties of Vc/C1 corneal neurons (excitation in response to cornea stimulation, inhibition by opioids) are common to other areas along the pain neuraxis and are consistent with a role of this region in the sensory-discriminative aspects of ocular pain. On the other hand, the heterogeneous responses of Vi/Vc corneal neurons to cornea stimulation and to opioids, together with the exclusive presence of neurons sensitive to the ocular moisture status, would suggest the involvement of this area in more specialized ocular functions, such as reflex control of tear formation and eye blinks, and in the recruitment of anti-nociceptive pathways. Consistently, single unit recordings from rat spinal trigeminal nucleus have identified in Vi/Vc two neuron types importantly involved in the initiation of the eye blink reflex (Henriquez and Evinger, 2007).

Second order neurons in Vi/Vc and Vc/C1 project to various brain regions including the bilateral parabrachial nuclear complex (PBN) (Cechetto et al., 1985; Panneton et al., 1994; Mitchell et al., 2004; Aicher et al., 2013; Aicher et al., 2014) and the posterior and medial contralateral thalamus (Dado and Giesler, 1990; Hirata et al., 2000; Guy et al., 2005; Saito et al., 2017). Other projections from Vi/VC and Vc/C1 neurons reach the periaqueductal gray (PAG), rostral ventral medulla, hypothalamus, and insular cortex (Bereiter et al., 2000; Sessle et al., 2000; Xiang et al., 2017). Recent anatomical and functional studies have reported that cornea stimulation activates a high number of Vc projection neurons directly targeting the PBN area, while ascending pathways to the thalamus seem to rely to more complex, polysynaptic circuits (Aicher et al., 2013; Aicher et al., 2014; Saito et al., 2017).

Neurons in PBN project to multiple brain regions, including central amygdala, hypothalamus, PAG, and ventrolateral medulla, which are considered to be involved in affective pain, autonomic and homeostatic control, and descending pain modulation (Gauriau and Bernard, 2002; Chiang et al., 2019). From the thalamus, information is sent to the somatosensory cortex (responsible for the sensory-discriminative aspects of pain) and to the limbic cortical areas (such as anterior cingulate cortex, insula and prefrontal cortex), involved in the affective/emotional components of pain. Brain imaging experiments performed on human subjects have described the “pain matrix” activated by ocular pain, which include numerous areas located in the cortices (insular, anterior cingulate, somatosensory and prefrontal cortex), in the thalamus, and in several subcortical centers (Moulton et al., 2009; Moulton et al., 2012; Tang et al., 2018).

### Mechanisms of Central Sensitization in Sp5

Similarly to peripheral terminals, also corneal nociceptor central terminals and Sp5 secondary neurons undergo plastic changes during long-lasting pain stimulation (Figure 2B). Peripheral sensitization, due to persistent inflammation or injury to the corneal nerve, and the subsequent increased afferent input to Sp5, can lead to central pain sensitization over time (Ebrahimiadib et al., 2020; Guerrero-Moreno et al., 2020). Evidence of central sensitization to corneal pain, expressed by an increased response of Sp5 neurons to cornea stimulation and the enlargement of cutaneous receptor fields, has been reported in rats in presence of endotoxin-induced uveitis (Bereiter et al., 2005), after corneal heating (Pozo and Cervero, 1993), in a model of photokeratitis (Tashiro et al., 2010), and after removal of exorbital gland (Rahman et al., 2015). In a model of cornea alkali burn, ERK phosphorylation (a marker of neuronal activation) was detected in mouse Vc/C1 and in higher brain areas belonging to the corneal neuropathic pain matrix (Xiang et al., 2017).

Beside neurons, central sensitization produces profound modifications also in Sp5 glial cells, as observed in various models of oro-facial pain: under trigeminal nerve injury, orofacial inflammation or migraine, several molecules are released from primary afferents, contributing to microglia and astrocyte activation (reviewed in Shinoda et al., 2019; Ye et al., 2021). Activated microglial cells and astrocytes release various pro-inflammatory cytokines (IL-1β, TNFα, and IL-6), chemokines (such as CCL-2), nerve growth factors (BDNF), and “gliotransmitters” (such as ATP, glutamate and peptides), that act on nearby glial cells and neurons, leading to an exacerbation of pain. In particular, astrocytes contribute to oro-facial pain central sensitization through several mechanisms: 1) increased phosphorylation of astrocytic Jun-N-terminal kinase (JNK) (Lin et al., 2019); 2) decrease of glutamate uptake, due to disfunction of the excitatory aminoacid transporter 2 (EAAT2) and/or of Na^+^/K^+^ ATPase pump (Isaksen et al., 2016; Zhou et al., 2019); 3) enhancement of synthesis and release of glutamine, a precursor of glutamate (Chiang et al., 2008); 4) increase of release of d-serine, a co-agonist of the NMDA receptor (Dieb and Hafidi, 2013); 5) potentiated function of astrocytic gap-junctions, which allow the propagation of calcium waves and the release of various gliotransmitters (Wang et al., 2014). A mechanism of microglia-astrocyte communication has been recently described: in the neuropathic pain model of infraorbital nerve injury, microglial cells release the complement component C1q, contributing to the activation of astrocytes in Sp5 and the induction of persistent orofacial pain (Asano et al., 2020).

Two recent studies indicate that glial cells play a critical role also in central sensitization to corneal pain. Ocular inflammation, induced in mice by topical instillations of benzalkonium chloride, was associated with microglia activation and enhancement of phosphorylated p38 MAPK specifically in these cells (Launay et al., 2016). Furthermore, upregulation of pro-inflammatory (IL-6, IL-1β), neuronal (ATF3, cFos) and glial (Iba1 and GFAP) markers was detected in both Vi/Vc and Vc/C1 in a mouse model of DED (Fakih et al., 2019). Interestingly, a higher immunoreactivity of the protein Piccolo (associated with the presynaptic zone and the secretion of synaptic vesicles) was also detected in the same study, suggesting a role of presynaptic plasticity in the sensitization of the nociceptive responses.

#### Glutamatergic Synaptic Transmission in Sp5 and Plasticity

In the spinal cord dorsal horn, pain sensitization is associated with maladaptive changes of the synaptic activity, which increase the efficacy of excitatory transmission and/or reduce the impact of synaptic inhibition (Gradwell et al., 2020). In the trigeminal nuclei similar mechanisms have been proposed, involving glutamatergic, GABA- and glycinergic transmission. Early electrophysiological studies had identified glutamatergic AMPA and NMDA receptors (AMPARs and NMDARs), together with GABA and glycine receptors, as the major synaptic receptor types involved in excitatory and inhibitory synaptic transmission in Sp5 (Hamba et al., 1998; Onodera et al., 2000; Takuma, 2001; Han et al., 2008).

The role of glutamate receptors in excitatory transmission at Sp5 and the intrinsic properties of neurons receiving these glutamatergic inputs have been extensively investigated in the superficial laminae (I- and II) of the subnucleus caudalis (Vc)**.** Vc lamina I neurons are considered to be both modality specific and WDR (wide dynamic range) (Renehan et al., 1986; Meng et al., 1997; Hirata et al., 1999). Neurons expressing the NK1 receptor are believed to represent projection neurons sending their axons to higher brain regions, similarly to what reported in spinal cord dorsal horn (Li et al., 2000; Sedlaceck et al., 2007; Luz et al., 2019). Neurons located in the Vc lamina II of cats and rats have also been functionally classified from *in vivo* recordings as WDR or nociceptor specific. Their electrophysiological characterization has revealed the presence of four different firing patterns: tonic, phasic, delayed, and single spiking (Davies and North, 2009). The use of fluorescent reporter mice allowed to correlate the tonic firing pattern prevalently to VGAT expressing GABA- and glycinergic neurons (inhibitory interneurons), while the delayed firing was most common in somatostatin/TdTomato neurons (predominantly excitatory interneurons), consistently with findings in spinal cord dorsal horn (Pradier et al., 2019).

Unmyelinated C and thinly myelinated, small-diameter Aδ primary afferent fibers are reported to be the major input to Vc laminae I-II neurons (Jacquin et al., 1986; Ambalavanar and Morris, 1992; Crissman et al., 1996). Excitatory synapses between nociceptive primary afferents and Vc neurons are primarily mediated by the activation of AMPARs and NMDARs (Grudt and Williams, 1994; Onodera et al., 2000).

New technical approaches, such as opto- and chemogenetics, are importantly contributing to the understanding of synaptic circuit organization in the spinal cord and trigeminal nuclei. In a recent study, optogenetic stimulation of primary afferent fibers, expressing TRPV1 and channelrhodopsin 2, evoked in mouse Vc glutamatergic mono- or polysynaptic responses, exhibiting different properties depending on the neuron type (Pradier et al., 2019). A similar experimental approach could be utilized also in the study of Sp5 synaptic circuits involved in corneal pain transmission. Early studies had demonstrated that the activation of such circuits strongly relies on glutamatergic receptors, since administration of AMPAR and NMDAR antagonists to Sp5 significantly decreased the c-Fos expression induced by corneal stimulation (Bereiter and Bereiter, 1996; Bereiter et al., 1996).

Glutamatergic receptors are critically involved in central sensitization in several forms of cranio-facial pain. NMDARs contribute to neuroplastic changes induced in adult rats by neonatal capsaicin treatment or by tooth pulp nociceptive stimulation (Chiang et al., 1997; Chiang et al., 1998). Dural application of an inflammatory soup (a model of migraine) enhanced phosphorylation of the NMDAR subunits NR1 and NR2B (Maneepak et al., 2009; Wang et al., 2018), while phosphorylation of the AMPAR subunit GluR1 is involved in neuron sensitization associated with dry tongue (Nakaya et al., 2016). Interestingly, release of SP from corneal CRs was increased in a mouse model of DED and the effect was mediated by sensitized TRPV1 channels (Li et al., 2019). SP release at the CR central terminals may amplify the excitation induced by glutamatergic transmission, leading to central sensitization and cold allodynia.

Several forms of synaptic plasticity, such as wind-up, long-term potentiation (LTP) and long-term depression (LTD), have been reported to contribute to central pain sensitization, requiring the activation of glutamate receptors (Sandkűhler and Gruber-Schoffnegger, 2012; Zhuo, 2017). In the Vc subnucleus, NMDARs play a critical role in the generation of wind-up, a form of short-term plasticity consisting of the increase in the total C-fiber mediated responses after repeated electrical stimulation (Luccarini et al., 2001; Woda et al., 2004). LTP can be generated in the same region by high frequency conditioning stimulation of C fibers: this mechanism, which seems to be mainly due to the activation of metabotropic glutamate mGluR5 receptors, could contribute to the persistent increase of neuron excitability observed in orofacial pain sensitization (Hamba et al., 2000; Liang et al., 2005). Recent experimental evidence has shown that LTD can be induced in Vc neurons following optogenetic stimulation of TRPV1-expressing nociceptive afferents and the subsequent activation of postsynaptic NMDARs (Pradier et al., 2018). Analogously to what observed in the spinal cord dorsal horn (Kim et al., 2015), a prevalence of LTD at synapses between primary afferent fibers and inhibitory neurons could be involved in disinhibition of synaptic circuits and increased sensitivity to nociceptive stimulation.

In an acute brain stem slice preparation, ascending and descending excitatory and inhibitory synaptic connections between Vi e Vc have been described, mediated by glutamate and by GABA or glycine, respectively. Interestingly, synaptic plasticity occurs also at these intersubnuclear connections: at Vi excitatory synapses, theta burst stimulation of ascending pathways from Vc generated LTD, that was converted in LTP in the absence of inhibitory transmission (Song and Youn, 2014).

#### Inhibitory Synaptic Transmission

As already mentioned, inhibitory transmission in Sp5 is mainly mediated by GABA, acting on both GABA_A_ and GABA_B_ receptors, and by glycine. Numerous studies have demonstrated that GABAergic and glycinergic neurons (about 30% of the total Vc neurons) inhibit Sp5 neuronal activity by both phasic and tonic activation (Grudt and Henderson, 1988; Matthews et al., 1988; Ginestal and Matute, 1993; Kondo et al., 1995; Takeda et al., 2000; Avendano et al., 2005; Han and Youn, 2008).

As shown by Hirata et al. (2003), corneal pain signaling in Sp5 is under strong GABAergic inhibition: microinjections of the GABA_A_ agonist muscimol into the Vi/Vc decreased nociceptive responses at Vc/C1, while local injections of muscimol at recording sites (at both Vi/Vc and Vc/C1) inhibited nociceptive transmission in all tested units.

Alterations in GABA- and glycinergic transmission play a key role in central pain sensitization. In the spinal cord dorsal horn, several mechanisms have been proposed for explaining the induction of disinhibition occurring during chronic pain. They include: 1) decrease of the number of GABA and glycinergic neurons; 2) reduction of GABA/glycine release and/or increased activity of their transporters; 3) decreased excitatory drive to inhibitory interneurons; 4) depolarization shift of chloride equilibrium potential (E_Cl_) in both primary afferent terminals and postsynaptic neurons (reviewed in: Gradwell et al., 2020; Comitato and Bardoni, 2021). Some of these mechanisms have been identified also in the trigeminal nuclei, contributing to synaptic disinhibition in chronic cranio-facial pain. Pharmacological blockade of GABA_A_ receptors (GABA_A_Rs) enhanced Sp5 neuron responses to orofacial mechanical stimulation, together with an expansion of receptive fields (Takeda et al., 2000). Following the transection of the inferior alveolar nerve, the number of neurons expressing the vesicular GABA transporter (VGAT) significantly decreased after seven days (Okada-Ogawa et al., 2015). Chronic constriction injury of rat infraorbital nerve (CCI-IoN) enhanced spontaneous activity of WDR neurons in Vc and decreased the tactile thresholds in all neurons. The development of mechanical allodynia was associated with a reduction of inhibition during paired-pulse stimulation and a decreased immunoreactivity to GAD65 (a marker of GABAergic neurons) (Martin et al., 2010). Similarly, CCI-IoN caused in Vc neurons the downregulation of two additional GABA neuron markers, GAD67 and parvalbumin*.* Intracisternal injections of vigabatrin, a blocker of the catabolic enzyme GABA transaminase, alleviated pain behaviour and restored normal GABA cell marker expression in allodynic Vc (Dieb and Hafidi, 2014).

Chloride equilibrium potential (E_Cl_) in primary afferent fibers and second order sensory neurons is set by the balance between the activity of two chloride transporters, NKCC1 (that accumulates Cl^−^ into the cell) and KCC2 (extruding Cl^−^). Upregulation of NKCC1 and/or downregulation of KCC2 causes the accumulation of Cl^−^ inside the neuron, a shift of E_Cl_ toward more depolarized potentials, and the conversion of GABA from inhibitory to excitatory transmitter (Guo and Hu, 2014; Comitato and Bardoni, 2021). Studies about modifications of chloride transporters in Sp5 under chronic pain conditions lead to controversial results. In the CCI-IoN model changes in E_Cl_ were modest and transient and did not persist during the late phase of neuropathic pain (Castro et al., 2017). In contrast, peripheral inflammation induced by a formalin injection into the vibrissa pad produced downregulation of KCC2, causing Cl^−^ accumulation inside Vc neurons (Wu et al., 2009). Similar effects have been obtained after transection of the inferior alveolar nerve in rats (Okada-Ogawa et al., 2015). These data indicate that alterations in the chloride transporter expression and function in trigeminal nuclei are heterogeneous and may depend on the pain model considered.

In spinal cord dorsal horn, GABA_A_Rs are involved in presynaptic inhibition of primary afferent terminals. The relative abundance of the NKCC1 transporter over KCC2 in dorsal root ganglion neurons sets their E_Cl_ value around -30 mV. Thus, opening of GABA_A_Rs on primary afferent central terminals causes a membrane depolarization that inactivates voltage-dependent channels and decreases glutamate release (Guo and Hu, 2014; Betelli et al., 2015). Since KCC2 mRNA is lacking in trigeminal primary neurons, a similar mechanism of presynaptic inhibition may occur also in trigeminal nuclei (Toyoda et al., 2005). Under chronic pain conditions, an increase of terminal depolarization, mediated by GABA_A_Rs, could turn presynaptic inhibition into facilitation, by inducing AP firing and increase of glutamate release. In the rat CCI-IoN model the upregulation of NKCC1 in TG primary neurons and the downregulation of KCC2 in Vc neurons were reported (Wei et al., 2013). This was associated with an excitatory action of GABA_A_Rs at both pre- and postsynaptic sites, leading to the increase of neuron excitability and possibly to presynaptic facilitation.

### Descending Modulation of Sp5: Role of Serotonin

Beside the GABA- and glycinergic system, an important role in synaptic inhibition of Sp5 neurons is exerted by descending modulatory pathways (Figures 2A,B). Second-order neurons in the Sp5 receive descending inputs from several regions of the central nervous system, such as the rostral ventral medulla or locus coeruleus, which modulate nociceptive and sensory inputs. RVM and the locus coeruleus, in turn, receive inputs from several brain regions, including amygdala, midbrain PAG, hypothalamus, and habenula. Descending modulation to trigeminal nuclei can be either inhibitory or facilitatory: while the inhibitory action is prevalent in physiological conditions, imbalance of inhibitory and facilitatory modulation in favour of facilitation, under tissue or nerve injury, can lead to chronic pain (Chung et al., 2020; Mills et al., 2020).

Descending facilitation is prevalently driven by the serotoninergic system, originating in the RVM from the nucleus raphe magnus (NRM) and its surrounding reticular formation, and projecting onto second-order neurons in trigeminal nuclei and spinal cord dorsal horn (Kwiat and Basbaum, 1992; Sugiyo et al., 2005; Okubo et al., 2013). Both Vi/Vc and Vc/C1 regions show dense serotoninergic innervation (Steinbusch et al., 1981) and receive projections from the NRM (Beitz, 1982). The involvement of serotonin in descending facilitation has been demonstrated in different models of oro-facial pain: mechanical hyperalgesia induced by masseter inflammation was relieved by the lesioning of RVM or by depletion of serotonin in RVM neurons (Sugiyo et al., 2005; Chai et al., 2012). In a recent study, chemogenetic silencing of RVM neurons, projecting to Vc, attenuated spontaneous and bite evoked pain in the same pain model (Chung et al., 2020). In the neuropathic CCI-IoN model, activation of serotoninergic receptors caused sensitization of TRPV1 channels and hyperactivity of TRPV1 positive afferent fibers (Kim et al., 2014).

Serotoninergic pain modulation in both spinal cord and Sp5 is achieved by activating heterogenous receptors (5-HTRs), ranging from 5-HT1 to 5-HT7 (Millan, 2002; Bardoni, 2019). Most of these receptors are G protein-coupled receptors, whereas only the 5-HT3 subtype is a cationic channel.

In naive animals, serotonin seems to exert a prevalent inhibitory action on Vc neurons: serotonin administration on mouse brainstem slices hyperpolarizes most neurons, by binding to 5-HT1(A) and 5-HT2 receptors (Yin, 2011). Furthermore, activation of 5-HT1R subtypes 1A and 1B/D, expressed on primary afferent terminals, inhibits glutamate release in rat brainstem slices (Jennings et al., 2004; Choi et al., 2013).

In pathological conditions, other 5-HTR subtypes seem to be involved in the facilitation of pain transmission, contributing to central sensitization in trigeminal nuclei. 5-HT2ARs, expressed on Vc PKCγ^+^ neurons (a subpopulation of excitatory interneurons), contribute to the development of inflammation induced mechanical allodynia by enhancing the density of synaptic spines (Alba-Delgado et al., 2018). Activation of 5-HT3Rs sensitizes TRPV1 receptors on central primary afferent terminals (Kim et al., 2014) and contributes to the maintenance of secondary hyperalgesia in a model of rat trigeminal nerve injury (Okubo, 2013). Finally, 5-HT7Rs induce the depolarization of a subpopulation of Vc neurons in the slice preparation (Yang et al., 2014), possibly increasing the excitability of Sp5 neurons under chronic pain conditions.

Serotoninergic modulation of cornea responsive units in Sp5 has been scarcely investigated. An *in vivo* study performed on rats has shown that NRM stimulation inhibits corneal evoked responses in Vi/Vc and Vc/C1 (Meng et al., 2000), confirming that transmission of corneal nociception in Sp5 is under control of the descending pathways. A recent study has described the involvement of habenular complex in the descending control of corneal pain: nociception induced by corneal application of saline can be inhibited by administration to habenula of morphine or lidocaine. Pre-treatment of the NRM with the 5-HT3 antagonist ondansetron prevented the effect of morphine on habenula, confirming the modulatory role played by this area on serotoninergic pathways (Khalilzadeh and Saiah, 2017).

## Concluding Remarks and Future Perspectives

During the last decades, numerous molecular biology, behavioural and electrophysiological studies have clarified several mechanisms occurring during corneal sensory transduction and peripheral pain sensitization. Morphological and electrophysiological analysis of trigeminal ganglion cells has also provided valuable insight about the functional properties of trigeminal primary neurons and their interactions with glial cells (Goto et al., 2016; Bista and Imlach, 2019).

Despite this progress, the characterization of the neural circuits and synaptic mechanisms involved in eye pain signaling at the spinal trigeminal nucleus is still largely incomplete. In the spinal cord, major advances have been obtained during the last decade in the understanding of the dorsal horn circuitry and plasticity. Using opto-and chemogenetic techniques, genetic labelling of neurons and advanced imaging technologies, it has been possible to selectively activate specific neuronal populations *in vitro* and *in vivo* and identify their role in somatic sensory transmission. In the brain stem, however, these high level technical approaches have been employed only very recently and many aspects of the synaptic network organization and function in trigeminal nuclei are still unknown.

As outlined in this review, persistent ocular pain produces both peripheral and central sensitization. Many questions still remain unanswered about the maladaptive changes occurring under chronic eye pain, especially those related to central sensitization in the Sp5 (Figure 1). First of all, the involvement of glutamate receptors in the different forms of synaptic plasticity (wind-up, LTP and LTD) during inflammatory and/or neuropathic corneal pain has not been investigated. The induction and maintenance of LTP at synapses with excitatory Sp5 neurons and/or LTD at inhibitory interneurons could play an important role in ocular pain sensitization.

Furthermore, results obtained in other models of cranio-facial pain suggest that a reduction in the efficacy of the GABA- and glycinergic inhibitory system may be critical also in chronic eye pain. However, the mechanisms responsible for Sp5 disinhibition in the different models of ocular pain still need to be clarified.

Finally, the role of descending modulation is not well defined: although a facilitatory role of serotoninergic pathways has been proposed in models of inflammatory and neuropathic eye pain, limited information is available about the circuits and receptors involved.

Based on these considerations, the acquisition of a better understanding of central processes mediating eye pain is an urgent need. A better knowledge of the cellular and molecular mechanisms involved in ocular pain sensitization will allow the identification of new players in pain transmission and the development of more effective pharmacological approaches, devoid of central side effects, for the treatment of the different forms of chronic ocular pain.

## Glossary

AITC: allyl isothiocyanate
AP: action potential
ASIC: acid-sensing ion channel
ATF-3: cyclic AMP-dependent transcription factor
BDNF: brain-derived neurotrophic factor
C1: upper cervical spinal cord
CCL-2: chemokine (C-C motif) ligand 2
CC-IoN: constriction injury of rat infraorbital nerve
CGRP: calcitonin-gene-related peptide
CR: cold-sensitive receptor
DED: dry eye disease
DRG: dorsal root ganglia
GAD67: glutamate decarboxylase 67
GFAP: glial fibrillary acidic protein
IBA1: ionized calcium binding adaptor molecule 1
IL: interleukin
MAPK: mitogen-activated protein kinase
MN: mechanonociceptor
NRM: nucleus raphe magnus
PAG: periaqueductal gray
PBN: parabrachial nucleus
PG: prostaglandin
PKC: protein kinase C
PN: polymodal nociceptor
RVM: rostral ventral medulla
SP: substance P
Sp5: trigeminal spinal nucleus
TG: trigeminal ganglion
TNF-α: tumor necrosis factor–alpha
TRPA1: transient receptor potential ankyrin 1
TRPV1: transient receptor potential cation channel subfamily V member 1
UV: ultraviolet radiation
Vc: subnucleus caudalis
VGAT: vesicular GABA transporter
Vi: subnucleus interpolaris
WDR: wide dynamic range
